# FEASIBILITY OF A NOVEL WEB-BASED NEUROPSYCHOLOGICAL REHABILITATION PROGRAMME FOR STROKE PATIENTS

**DOI:** 10.2340/jrm.v57.43275

**Published:** 2025-09-23

**Authors:** Outi VUORI, Heidi WIK, Annamaria WIKSTRÖM, Hanna JOKINEN, Marja HIETANEN, Eeva-Liisa KALLIO

**Affiliations:** 1 Division of Neuropsychology, HUS Neurocenter, University of Helsinki and Helsinki University Hospital, Helsinki; 2 Department of Psychology, Faculty of Medicine, University of Helsinki, Helsinki, Finland

**Keywords:** cognition, neuropsychology, psychoeducation, rehabilitation, stroke, telerehabilitation, web-based rehabilitation

## Abstract

**Objective:**

To evaluate the implementation and feasibility of the Neuropsychological Digital Care Pathway (NP-DCP), a novel, professional-guided web-based rehabilitation programme for the rehabilitation of cognitive and emotional symptoms following stroke and to identify factors associated with adherence and user experience.

**Design:**

Retrospective registry study.

**Subjects:**

133 stroke patients (≥ 18 years) with mild to moderate cognitive impairment at the Neurocenter, Helsinki University Hospital, Finland, referred to the NP-DCP between April 2019 and April 2020.

**Methods:**

The NP-DCP adherence data (completers, dropouts, non-starters) and feedback from programme completers were analysed along with demographic and clinical characteristics.

**Results:**

Of the referred patients, 114 (86%) started the programme; of these, 79 (68%) completed it. The average completion time was 82.3 days. Referral on discharge from acute care was associated with higher adherence (*p* = 0.019). Women were more likely to start the programme than men (*p* = 0.012). Usability and content were rated good to excellent (mean 4.1–4.4/5), with participants with basic education more satisfied than those with higher education (*p* = 0.033).

**Conclusion:**

The NP-DCP demonstrated high engagement and user satisfaction, suggesting it is a feasible tool for stroke rehabilitation, thus adding a promising new method to existing services. Early referral may improve adherence.

## LAY ABSTRACT

A new web-based neuropsychological rehabilitation programme, the Neuropsychological Digital Care Pathway, was developed and evaluated to help post-discharge stroke patients manage their mild to moderate cognitive symptoms. It was designed to be carried out independently, with remote professional guidance. This registry study comprised 133 participants with stroke aged ≥ 18 who were referred to the programme between April 2019 and April 2020. Among the participants, 86% started the programme, 68% of whom completed it. The programme took an average of 82.3 days to complete. Higher adherence was linked to early referral to the Neuropsychological Digital Care Pathway after discharge from acute stroke care. The programme was considered useful, and patients gave positive feedback, with most rating their experience as good or excellent. Overall, the Neuropsychological Digital Care Pathway shows promise as a feasible and valuable tool for stroke rehabilitation and can complement existing rehabilitation services.

Over 12.2 million new strokes occur annually, with 62% affecting people under 70 years (1). Post-stroke cognitive impairment is common (2), often involving memory, attention, and executive functions (3–5). These impairments hinder recovery and, even in mild strokes, predict poorer functional outcomes, including a reduced ability to return to work (4–7). While most cognitive improvements occur within 6 months (7), nearly half of young stroke patients experience persistent cognitive impairments into chronic phases (8). Despite this, 22–40%, even of the working-aged patients, particularly those with mild symptoms, do not receive any rehabilitation after acute care (5). Post-stroke follow-up interventions typically focus on medical secondary prevention rather than comprehensively addressing cognitive and psychological needs (9).

Neuropsychological rehabilitation aims at restoring and compensating for the cognitive, emotional, and behavioural sequelae of acquired brain injury, supporting reintegration into daily activities and improving outcomes through psychoeducation, skills training, and strategies for compensation and emotional regulation (10, 11). Moreover, patients often face emotional distress in early stages of recovery, and timely neuropsychological rehabilitation enhances functional and emotional outcomes, promoting their independence and quality of life (10). However, access to neuropsychological rehabilitation remains limited in many regions due to unevenly distributed resources, as it relies on face-to-face delivery (5, 12).

Limited rehabilitation resources and advancing technology drive the need for telerehabilitation methods to complement traditional interventions and ensure effective care. Telerehabilitation improves accessibility by enabling rehabilitation regardless of time and location, thus benefiting patients in remote or underserved areas (13). Studies indicate that telerehabilitation services can also be cost-beneficial, allocating professionals’ limited time to more patients than usual (14, 15). However, the approach is pioneering, and challenges, like low adherence and dropout rates, remain (15, 16).

Evidence supports the feasibility and acceptability of web-based rehabilitation across various settings and patient populations, including individuals with psychiatric conditions, where web-based psychotherapy is widely used (13, 16–21). For example, the *Remind* programme demonstrated feasibility for postoperative cognitive rehabilitation in brain tumour patients (17, 22), while a digital care pathway for multiple sclerosis patients showed high satisfaction and psychological benefits (23). In stroke care, existing mobile and web-based applications generally target specific functions, such as cognitive or motor training (24, 25), with limited evidence on their clinical usability and outcomes (26).

A structured web-based rehabilitation programme, the Neuropsychological Digital Care Pathway (NP-DCP), was developed for stroke patients at Helsinki University Hospital, Neurocenter, based on the approaches that have proved effective in neuropsychological rehabilitation. These include in particular psychoeducation (27, 28), compensative strategy training for attention, executive functioning, and memory (10, 11, 29), and metacognitive strategy training with a supportive framework (10). The programme was designed for independent use, with remote neuropsychologist support to address questions and promote adherence, as professional guidance has been shown to improve it (30).

The study has 3 goals: (*i*) to describe the development and implementation of a web-based intervention, NP-DCP, for stroke rehabilitation, (*ii*) to evaluate the accessibility, usability, and user experiences of the programme, and (*iii*) to identify the factors associated with adherence and user experience.

## METHODS

### Study design

Participants for this retrospective cohort study were sourced from real-world hospital treatment registries at HUS Helsinki University Hospital. Participants comprised a consecutive series of adult stroke patients referred to the NP-DCP from April 2019 to April 2020. This registry-based study was conducted without any direct contact or communication with the participants.

The study was approved by the HUS medical ethics review board (HUS 1393/2021, 3/15/2023). The research project was conducted in accordance with the principles of the Declaration of Helsinki. The data processing practices followed the European Union Data Protection Directive Rules.

### Development and use of the programme

The NP-DCP, a web-based neuropsychological rehabilitation programme, was developed on the *Health Village* platform (www.terveyskyla.fi/en) at the Neurocenter of HUS Helsinki University Hospital in 2018 and 2019. *Health Village* is a public online service jointly developed by university hospitals in Finland (Helsinki, Tampere, Turku, Kuopio, and Oulu), with content created by specialized healthcare professionals in collaboration with service users. The My Path (Omapolku) channel on *Health Village* (www.terveyskyla.fi/en/mypath) provides digital care pathways for patients with a referral. It is a secured service requiring strong authentication.

The NP-DCP was built on evidence-based data and current clinical practices of neuropsychological rehabilitation utilizing psychoeducation, skills training, and strategies for the compensation of cognitive deficits and emotional distress (10, 11). Designed for adult Finnish-speaking stroke patients with mild to moderate cognitive deficits, the programme was piloted between December 2018 and April 2019. Based on the feedback from pilot participants and clinical neuropsychologists, refinements were made before its formal incorporation into HUS clinical practice in April 2019.

The referral criteria for the NP-DCP programme are as follows: (a) < 12 months from stroke occurrence; (b) mild to moderate cognitive symptoms as evaluated by a professional (e.g., physician, neuropsychologist, occupational therapist) or subjective cognitive complaints following stroke; (c) proficient in Finnish; (d) age 18 years or over; and (e) the ability and willingness to use digital appliances. The exclusion criteria include the following: (a) progressive neurological condition affecting cognition; (b) severe or acute psychiatric disorders affecting cognition and compliance; or (c) current complex substance use disorder.

**Fig. 1:**
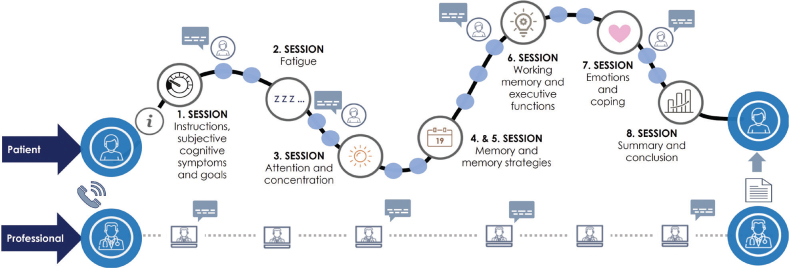
Schematic presentation of the NP-DCP. In this structured rehabilitation model, patients progress independently through 8 sessions (upper line), while healthcare professionals oversee their progress and provide support via a secure messaging channel when necessary (lower line). Prior to initiating the NP-DCP, a professional contacts patients via telephone to provide guidance on accessing the digital service platform. Upon completion of the programme, the professional writes a summary report detailing the patient’s engagement and the content delivered throughout the intervention.

Patients access the NP-DCP by logging onto the My Path service channel with secure e-identification. They progress independently under the supervision of a neuropsychologist through the programme at their own pace regardless of time and location, with an option to contact a neuropsychologist via a messaging channel. Neuropsychologists reply within 3 business days and, when needed, provide feedback on the patient’s progress. The NP-DCP comprises 8 sessions, which are mandatory to complete in a specified order (Fig. 1). A minimum of a 3-day interval between sessions is enforced to allow time to rehearse and integrate session practices into daily life. If a patient has not logged on for 7 days, an automated reminder is sent, followed by a personalized message from a neuropsychologist if inactivity persists. The NP-DCP is free of charge for the user and is offered by the national public healthcare system.

### Content of the programme

The NP-DCP provides patients with information on common cognitive impairments post-stroke and strategies for managing symptoms in daily life. Programme content and session themes are summarized in Table I. Each session comprises 12–14 tasks, incorporating texts, images, videos, and information links to explain cognitive symptoms and their management. Depending on patient engagement, sessions take approximately 1–2 h to complete. Personal goal setting is encouraged to enhance motivation, while self-awareness is fostered with symptom questionnaires, automated feedback, and self-reflection exercises. Virtual peer support is provided through case examples featuring fictional neurological patients of various ages.

**Table I:** Content of the Neuropsychological Digital Care Pathway

Session subject	Session introduction
1. Welcome	In the first session, patients are told what online independent rehabilitation is and how to use the service and its message channel. Topics related to recovery include the brain, cognitive functioning, and changes in it. Patients fill out a subjective symptom questionnaire and receive automated feedback, name personal goals for recovery, and reflect on life changes related to their illness. Patient also get to know peer stories and fill out a questionnaire considering everyday activity, need for sleep, fatigue, and mood. The questionnaire is filled out in every session and allows patients to monitor the progress of their recovery
2. Fatigue	The second session focuses on fatigue and its management. Patients are told that fatigue faster than usual is a common consequence of brain disease or injury, and equally common is the relief of fatigue as recovery progresses. The session discusses time management and lifestyles that support their daily coping with fatigue. The techniques practised are relaxation and mindfulness. In addition, the personal goals for recovery are reviewed again
3. Attention and concentration	Patients are informed how to consciously support and develop the maintenance of attention and concentration. Patients are guided to do a concentration exercise. The third session also discusses lifestyles that support brain health, such as abstinence and exercise
4. Memory	In the fourth session, different aspects of memory, memory problems associated with brain diseases and injuries, and everyday memory support methods are reviewed and practised. Patients can evaluate and are encouraged to try out memory-supporting techniques in their own everyday life
5. Memory strategies	The fifth session will continue working on the memory topic. The session starts with a short memory test, and after that the patient continues to practise memory skills (for example spaced retrieval and internal aids, such as chunking, acronyms, face–name mnemonics, and visualization)
6. Working memory and planning	Working memory is discussed in the sixth session. The topics also includes executive functions and especially the planning of one’s own actions. A spouse or another person close to a patient can also fill out a questionnaire evaluating the patient’s executive skills, if the patient so wishes. Patients are instructed to do a planning task at home
7. Emotions	In this session, thoughts and feelings related to sudden illnesses or accident are reviewed, and patients learn how disturbingly emotional reactions can be tolerated and regulated. Patients can also reflect more closely on their life values and future goals. Finally, patients take a memory test, the items of which will be asked about at the following session
8. Summary	In the last session, patients get to test their memory skills one more time in the memory test given in the previous session. This session summarizes the treatment and focuses on the time after the treatment period. Also the session informs patients how they can promote their recovery in the future. At the end of the programme, a neuropsychologist adds a short summary of the rehabilitation period to the patient’s treatment path

### Participant selection

**Fig. 2:**
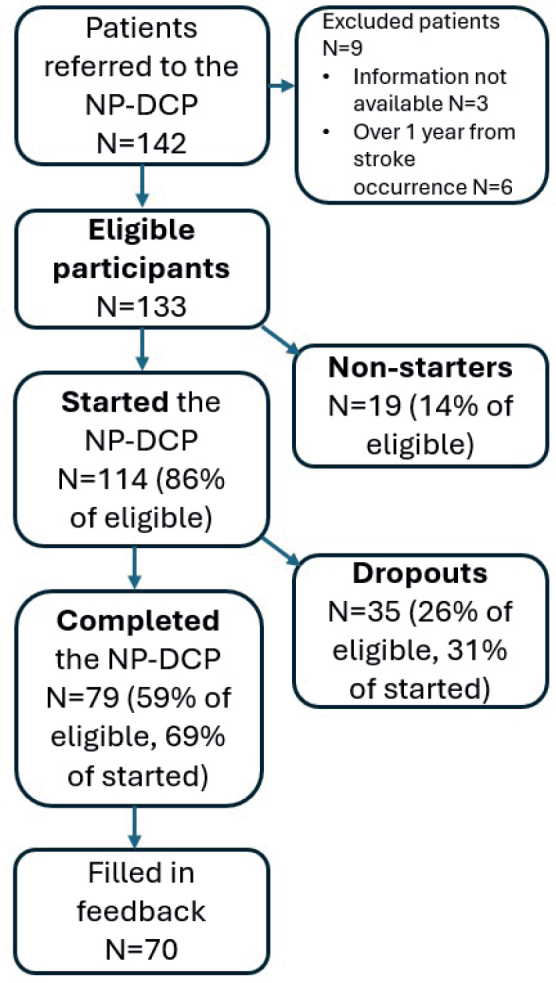
Flowchart of the participant selection for the study and adherence rates in the Neuropsychological Digital Care Pathway (NP-DCP).

Participant selection for the study is described in Fig. 2. We identified 142 patients who were referred to the NP-DCP during the study period, April 2019 to April 2020. Patients were referred to the NP-DCP by their physicians (neurologist or another specialist) across Finland, based on a clinical neuropsychologist’s assessment and recommendation. However, 6 patients did not fulfil the inclusion criteria for the programme (i.e., less than 1 year from stroke), and medical information was not available for 3 patients, who were residents outside the HUS area. Therefore, we received a total of 133 eligible participants in this study.

### Data collection

The demographic and clinical patient data were gathered from medical records in Apotti patient registries (Epic Systems Corporation), user engagement data from digital care pathway (DCP) technical registries, and patient-reported outcomes (feedback questionnaire) electronically from the NP-DCP platform. For patients with a neuropsychological assessment as part of routine care, original test sheets were requested from the medical archive when necessary. All the data was collected post-stroke and approximately 3 years after completing the NP-DCP (in July 2023). Patient data from the different HUS registries were linked using the patient’s social-security identification (ID) number and later anonymized with a research ID.

## MEASURES

### Participant characteristics

Demographic data included age, sex, education level, and employment status. In addition, clinical data comprised the stroke type, modified Rankin Scale (mRS) score for measuring the degree of functional disability after discharge (31) (if not noted in the epicrisis, scaled retrospectively from medical records), duration of acute stroke care, length of sick leave, details of post-stroke follow-up care, and details of neuropsychological care, rehabilitation, and follow-up. The data also included the timing of referral to the NP-DCP.

Most participants (*n* = 127, 95%) had been assessed by a neuropsychologist prior to their referral to the NP-DCP as part of their standard care during the acute or subacute phase of their stroke recovery. The severity and affected cognitive functions were collected from neuropsychologists’ clinical assessment, as documented in medical records, to describe the participant characteristics considering the level of cognitive symptoms before referring to the NP-DCP.

### User data on the NP-DCP

User data from the NP-DCP were extracted from the My Path service, including the starting and ending dates of the programme, NP-DCP session completion, and messages exchanged between users and professionals (neuropsychologists). Observations on the use and engagement of NP-DCP are based on the participants who initiated or both initiated and completed the NP-DCP. Participants who completed Session 7 (approximately 85% of the programme) were classified as completers, while those who started but did not complete were deemed dropouts. Participants who were referred but did not initiate the programme were designated as non-starters. Considering the retrospective nature of this study, reasons for non-starting or dropout could not be recorded; however, demographic and clinical information on this participant group will be reported.

### User experience

After completing the programme (Session 8), participants were asked to fill in a questionnaire evaluating their experiences with the NP-DCP (Table II). The feedback questionnaire was formulated by the content creators to determine what content the users evaluated as most useful and how satisfied they were with the content. This user feedback was implemented at the end of the NP-DCP programme and collected digitally through the My Path service.

**Table II:** User feedback questionnaire on the Neuropsychological Digital Care Pathway

Patient satisfaction 1 = Strongly disagree, 2 = Disagree, 3 = Undecided, 4 = Agree, 5 = Strongly agree	Mean	How much did the following programme content help you? 1 = Not at all, 2 = To a small extent, 3 = To a moderate extent, 4 = To a large extent, 5 = To a very large extent	Mean
I have enough knowledge, considering my condition and rehabilitation	4.5	Knowledge regarding neuropsychological symptoms	4.4
Contacting a neuropsychologist was easy during the programme	4.0	Knowledge and ways to manage fatigue	4.1
The programme was easy to use	4.5	Knowledge and ways to support attention and concentration	4.2
The programme was useful	4.5	Knowledge and ways to support memory	4.2
The programme helped me during my recovery	4.1	Practising different memory strategies	4.1
I would recommend the programme to a friend with the same condition	4.6	Knowledge and ways to enhance executive functions	3.9
Overall	4.4	Knowledge and ways to regulate and control emotions	3.9
		Setting goals for recovery	4.1
		Overall	4.1

### Statistical analysis

Descriptive statistics of participants were presented, including the proportions of eligible, included, excluded, dropout, and completing patients. Group comparisons were conducted using Pearson’s χ2 test or Fisher’s exact test for categorical variables, an independent samples *t*-test for ordinal sum variables, and one-way ANOVA for continuous variables with Tukey’s test for pairwise comparison, if applicable. All the data collection and statistical analysis took place in the secure IT environment of HUS.

## RESULTS

### Characteristics of the participants

Table III presents the demographic, clinical, and NP-DCP characteristics of the participants. Participants’ ages ranged from 21 to 79 years (M = 50.0, SD 11.8), with 41% identified as female. The majority of participants (93%) were residents from the HUS area (of which Helsinki comprised 50%), with 7% living in other areas of Finland. Among the eligible population, 92% were gainfully employed and 8% were not including students (2%), unemployed individuals (3%), and pensioners (4%).

**Table III:** Demographic, clinical, and Neuropsychological Digital Care Pathway characteristics in all participants and in 3 adherence groups

Characteristic	All participants (*n* = 133)	Completers (*n* = 79)	Dropouts (*n* = 35)	Non-starters (*n* = 19)	Group comparison	
					Test statistics	*p*-value
Age (years), mean (SD)	50.0 (11.8)	50.7 (11.9)	49.3 (11.9)	48.5 (11.6)	ANOVA	0.725
Sex *n* (%)					χ2(2)	0.012
Female	55 (41%)	36 (46%)	17 (49%)	2 (11%)		
Male	78 (59%)	43 (54%)	18 (51%)	17 (89%)		
Education					χ2(4)	0.156
Primary education	16 (13%)	6 (8%)	6 (18%)	4 (22%)		
Upper secondary	45 (35%)	26 (35%)	15 (44%)	4 (22%)		
Higher education	66 (52%)	43 (57%)	13 (38%)	10 (56%)		
Diagnosis					χ2(2)	0.350
Ischaemia	116 (89%)	69 (90%)	32 (91%)	15 (79%)		
Haemorrhage	15 (11%)	8 (10%)	3 (9%)	4 (21%)		
mRS					χ2(4)	0.492
≤ 1	72 (55%)	47 (62%)	16 (46%)	9 (47%)		
2	32 (25%)	17 (22%)	10 (29%)	5 (26%)		
≥3	26 (20%)	12 (16%)	9 (26%)	5 (26%)		
Severity of cognitive symptoms prior to the NP-DCP					χ2(2)	0.282
Mild	81 (64%)	52 (69%)	20 (57%)	9 (53%)		
Moderate/severe	46 (36%)	23 (31%)	15 (43%)	8 (47%)		
Care after acute stroke treatment					Fisher’s exact	0.033
Home, neurological/neuropsychological follow-up	69 (53%)	45 (58%)	12 (34%)	12 (63%)		
Outpatient rehabilitation (neuropsychological)	43 (31%)	24 (31%)	13 (37%)	3 (16%)		
Inpatient rehabilitation (any)	22 (17%)	8 (10%)	10 (29%)	4 (21%)		
Neuropsychological rehabilitation					χ2(6)	0.071
Only the NP-DCP	55 (43%)	33 (44%)	12 (34%)	10 (56%)		
Brief counselling (1–5 visits)	34 (27%)	19 (25%)	11 (31%)	4 (22%)		
Outpatient rehabilitation	25 (20%)	19 (25%)	4 (11%)	2 (11%)		
Inpatient rehabilitation	14 (11%)	4 (5%)	8 (23%)	2 (11%)		
Time of referral					Fisher’s exact	0.019
After acute care discharge	70 (53%)	50 (63%)	15 (43%)	5 (26%)		
Outpatient neuropsychological examination (> 3 months after stroke occurrence)	35 (26%)	18 (23%)	10 (29%)	7 (37%)		
During outpatient rehabilitation or discharge from inpatient rehabilitation (neuropsychological)	28 (21%)	11 (14%)	10 (29%)	7 (37%)		
Time from stroke occurrence to starting the NP-DCP days (SD)	58.9 (66.1)	48.8 (59.7)	72.2 (78.2)	75.0 (62.3)	ANOVA	0.114
Length of sick leave days (SD)	202.8 (202.5)	174.5 (139.3)	226.9 (194.6)	269.1 (362.7)	ANOVA	0.150
Return to work					Fisher’s exact	0.735
Full-time	42 (35%)	23 (33%)	12 (36%)	7 (44%)		
Part-time	41 (35%)	27 (39%)	11 (33%)	3 (19%)		
Vocational rehabilitation	13 (11%)	8 (11%)	4 (12%)	1 (6%)		
Retirement	23 (19%)	12 (17%)	6 (18%)	5 (31%)		

Bold *p*-values indicate significant at *p* < 0.05.

Based on clinical neuropsychological assessments during hospitalization before referral to the NP-DCP, the most commonly impaired cognitive functions following stroke were attention (71% of participants assessed by a neuropsychologist), memory (66%), and executive functions (46%). Table IV presents the mean cognitive test results of the study group with standard deviations. Cognitive deficits were classified by a clinical neuropsychologist as mild in 81 (64%) participants, moderate in 39 (31%), and severe in 7 (6%) participants at the time of referral. Moderate and severe symptom groups were combined for the statistical analysis due to the small group size of the severe symptom group. Some 21% of the participants were reported to have fatigue after stroke.

**Table IV:** Neuropsychological test results prior to the Neuropsychological Digital Care Pathway

Test	*n*	Mean (SD)
Logical memory (WMS-R), raw scores (max 50 points)	84	22.4 (7.4)
Logical memory (WMS-R), delayed, raw scores (max 50 points)	89	20.2 (7.2)
Word list, 10 words x 4 (max 40 points)	108	30.2 (5.4)
Word list, delayed (max 10 points)	108	6.8 (2.3)
Word fluency, words starting with letter K per minute	115	16.6 (6.7)
Word fluency, animals per minute	122	19.5 (6.9)
TMT-A, time (s)	124	46.4 (24.9)
TMT-B, time (s)	125	113.5 (65.1)
Finger tapping in 10 s, dominant	98	51.9 (9.1)
Finger tapping in 10 s, non-dominant	98	47.0 (10.1)

WMS-R: Wechsler Memory Scale Revised (32); TMT-A: Trail Making Test part A; TMT-B: Trail Making Test part B (33).

### Accessibility and use of the NP-DCP

All the participants used the programme independently, living in the community after discharge from hospital care. After acute stroke care, most of the participants (53%) were discharged home with a neurological or neuropsychological follow-up but no neuropsychological or other types of rehabilitation; 31% had outpatient neuropsychological rehabilitation, and 17% were referred to inpatient rehabilitation after acute stroke care. Among all participants, 53% were referred to the NP-DCP after acute care discharge, 26% after outpatient neuropsychological examination (> 3 months after stroke occurrence), and 21% during outpatient neuropsychological rehabilitation or discharge from inpatient neuropsychological rehabilitation.

The average delay in starting the NP-DCP from adding patients to the platform was 12.5 (33.5) days. The programme was logged into 1,443 times during the study period. On average, eligible patients logged in 11.8 times per treatment period and completed 8.4 tasks per week. The NP-DCP took 82.3 (39.8) days to complete on average. The number of messages exchanged within the programme varied: 89% of patients sent 0–1 message, with only 9 patients sending 2 or more messages (min–max 0–15).

### User engagement with the NP-DCP

The adherence rates are presented in Fig. 2. In all, 86% of the eligible patients started the NP-DCP and 14% were non-starters. The completion rate was 59% among eligible patients and 69% of those who started the programme. The overall dropout rate was 26% among eligible patients. Dropout patients completed approximately half of the programme (*Mdn* = 4.12, min–max 1.1–7.11 sessions).

Participants who did not start the programme were more often men (17 men vs 2 women, χ²(2, *n* = 133) = 8.8, *p* = 0.012). No other significant differences were found between the groups in terms of demographic characteristics (age, education, or employment status).

There was a significant association between the care after acute stroke treatment and the adherence group (*p* = 0.033) (see Table III). Among completers, 58% were discharged without rehabilitation, compared with 63% of non-starters and 34% of dropouts. Neuropsychological outpatient rehabilitation was received by only 16% of non-starters, whereas 31% of completers and 37% of dropouts received such care. In contrast, inpatient rehabilitation was provided to just 10% of completers, while 21% of non-starters and 29% of dropouts received inpatient services.

A significant association was found between the time of referral and adherence group (*p* = 0.019) (see Table III). Among completers, 63% were referred to the NP-DCP following discharge from acute care, compared with 43% of dropouts and 26% of non-starters. Conversely, 37% of non-starters were referred during outpatient rehabilitation or on discharge from inpatient rehabilitation, compared with 14% of completers and 29% of dropouts. No significant differences emerged between the completers, dropouts, and non-starters regarding deficits in cognitive functions or neuropsychological test results (Table IV and Table SI). Of those who completed the programme and provided feedback, 68% had mild cognitive symptoms, while 32% had moderate or severe symptoms.

### User experience

The results of the user experience questionnaire are listed in Table II. Overall, user satisfaction was rated 4.4/5 and the content 4.1/5. Users considered the NP-DCP easy to use and useful (4.5/5) and stated that they would recommend it to someone with the same condition (4.6/5). Users experienced receiving knowledge regarding their condition and rehabilitation (4.5/5) and neuropsychological symptoms (4.4/5). There was a significant association between user satisfaction and education [F(2, 147) = 4.32, *p* = 0.017]. Patients with basic education were more satisfied with the NP-DCP than patients with higher education (*p* = 0.033). No significant associations were found between the severity of cognitive symptoms and patient feedback. Additionally, neither age nor sex-based differences were observed in the feedback.

## DISCUSSION

Due to limited neuropsychological rehabilitation services and insufficient resources, telerehabilitation has the potential to address the shortage in cognitive rehabilitation, although the field is still new and underdeveloped (5, 12, 26). The Neuropsychological Digital Care Pathway (NP-DCP), developed at the Neurocenter, Helsinki University Hospital, is one of the first structured, independently used but professional-guided, neuropsychological web-based rehabilitation programmes for post-stroke cognitive impairment and emotional distress. This retrospective registry study aimed to evaluate the programme’s acceptability and usability, as well as factors associated with adherence. Background, clinical, and NP-DCP data were gathered and analysed.

In general, the NP-DCP was highly accepted and usable as a rehabilitation tool among stroke patients. Patients committed to the programme and rated its content as a valuable source of reliable information following stroke. They also found the NP-DCP highly recommendable for others with the same condition. Completion rates were highest among patients with mild cognitive symptoms, who were referred shortly after acute care, and had no concurrent rehabilitation.

The NP-DCP appeared accessible to a wide range of stroke patients, with participants ranging from 19 to 79 years of age and residing across Finland. In this study population, all users completed the programme independently, whereas in many previous telerehabilitation studies patients used the programme with the support of a caregiver (13, 24). The mean age of the participants was higher than reported in previous digital interventions (15, 21, 23), suggesting suitability for older users as well. Participants engaged with the NP-DCP at various stages of recovery, varying from early referral after acute care discharge to a maximum of 1 year from the initial occurrence and also from no other rehabilitation to multidisciplinary inpatient rehabilitation. Given the uneven access to traditional neuropsychological rehabilitation, these findings suggest that the NP-DCP may provide a more accessible and flexible alternative, regardless of patients’ place of residence or age (12, 13).

Adherence to the NP-DCP was high. The percentage of non-starters, 14%, is comparable to or lower than those reported in other web-based intervention studies for patients with mood disorders, neurological patients, and healthy participants, where non-starter rates range from 36% to 55% (15, 19, 26, 34, 35). More than two-thirds of the patients who started the programme completed it, representing a favourable outcome for a largely self-directed rehabilitation intervention. Comparison is complicated by the unclear reporting of dropout rates in many prior studies, possibly because of heterogeneity in study settings and programme formats. However, the dropout rate of this study’s clinical group is comparable to a recent study of brain tumour patients (22).

The timing of referral appeared to be a factor associated with programme adherence. Early referral to the NP-DCP, particularly of those discharged home without outpatient or inpatient rehabilitation, was associated with greater programme adherence. This finding addresses the recognized need for post-acute support and rehabilitation following stroke (5, 9–11) contrasting with studies where the early-phase use of independent rehabilitation was associated with only modest adherence (17, 36). This highlights the importance of recognizing individual rehabilitation needs early after stroke (6, 7).

Adherence to the NP-DCP varied according to post-acute care. Patients who underwent inpatient rehabilitation were less likely to complete the programme, consistent with the limited added value of combining web-based rehabilitation with conventional rehabilitation (36). Although adherence was not significantly associated with cognitive symptom severity, this finding might still indicate that patients in inpatient rehabilitation tend to have more severe symptoms than those discharged after acute stroke care. Interestingly, patients discharged without further rehabilitation were overrepresented among both completers and non-starters, suggesting a distinction between those motivated to engage with the programme and those who did not perceive the need for it. In a prior study, the absence of subjective cognitive complaints may have contributed to lower adherence (17). The referral criteria of the NP-DCP did not distinguish between subjective and objective cognitive complaints. Given the mixed evidence on how symptom severity relates to adherence (34), objective clinical symptoms and patients’ subjective experiences should be considered when referring to digital rehabilitation. Finally, men were less likely to start the programme, consistent with lower compliance in men in stroke and web-based treatments (34, 37).

Overall, user experiences with the NP-DCP were positive, aligning with high satisfaction rates reported for other web-based programmes (13, 15, 22, 23). Notably, patients found the NP-DCP helpful in providing reliable information on post-stroke cognitive symptoms, recovery, and symptom management, all of which are areas where support remains limited (9, 26). Although overall user satisfaction was high, patients with basic education were more satisfied than those with higher education, somewhat contrasting with previous studies, where higher education is linked to a greater acceptance of telerehabilitation (38). Participants’ levels of education were generally high but reflected those of the broader population in Finnish and Helsinki areas (39).

### Strengths and limitations

The main strength of the study is the inclusion of a consecutive series of patients referred to the NP-DCP over 1 year, minimizing the selection bias common in self-selected intervention studies. Notably, this is one of the few studies to include data on clinical patients who opted not to start the programme. However, the retrospective design limited our ability to explore reasons for non-initiation or dropout, and the absence of a control group restricts causal conclusions regarding the programme’s effectiveness. In addition, concurrent participation in other rehabilitation was not an exclusion criterion in this study. Although this was controlled, concurred rehabilitation still might have influenced the adherence, user behaviour, or feedback. Despite the use of a clinically representative sample, the results are not generalizable to all stroke patients due to the referral criteria, which focused specifically on individuals with mild to moderate cognitive symptoms. Additionally, feedback was obtained only from the programme completers, potentially biasing positive evaluations. Furthermore, the study timeline coincided with the COVID-19 pandemic, leading to the referral of more severe patients to the NP-DCP than recommended, as face-to-face rehabilitation was partly restricted in Finland, possibly increasing the non-completion rates.

Most participants had relatively mild cognitive symptoms and a low degree of disability, consistent with the referral criteria of the programme. Traditional face-to-face neuropsychological rehabilitation is often reserved for patients with more severe symptoms due to limited resources (5), whereas web-based programmes extend services to those with milder impairments. This study offers valuable information on a novel rehabilitation method, which can be implemented despite limited rehabilitation resources. We could not calculate the cost-effectiveness in this retrospective registry study; however, clinical neuropsychologists likely spend less time supporting patients in web-based programmes than in face-to-face rehabilitation (14). Web-based programmes are easily accessed and could diminish regional differences in rehabilitation resources (12, 13). Moreover, web-based interventions have the advantage of bypassing waiting lists and enabling the immediate initiation of rehabilitation.

In conclusion, the NP-DCP programme represents a promising new tool in the field of telerehabilitation for neurological patients. The study demonstrates the feasibility and acceptability of a structured, professional-guided web-based programme as a rehabilitation method for adult stroke patients, particularly for those with mild cognitive symptoms and in the early stages of recovery. Patients were able to engage with the programme independently and reported it as beneficial. This type of intervention may also help reduce regional disparities in access to rehabilitation services. However, further research is needed to determine the clinical effectiveness of web-based rehabilitation and to support evidence-based recommendations for the implementation in practice.

## Supplementary Material


