# Supplementary material for: FEASIBILITY OF A NOVEL WEB-BASED NEUROPSYCHOLOGICAL REHABILITATION PROGRAMME FOR STROKE PATIENTS
Source: J Rehabil Med. 2025 Sep 23;57:43275. doi: 10.2340/jrm.v57.43275 (PMC12476052; doi:10.2340/jrm.v57.43275)
Supplement: Supplementary file 1 [file JRM-57-43275-s1.pdf]

Supplementary material has been published as submitted. It has not been copyedited, or typeset by Journal of Rehabilitation Medicine

Table SI. Comparison of affected cognitive function and neuropsychological test results in different adherence groups prior to the NP-DCP

**Impaired cognitive domain**	Completers	Drop-outs	Non-starters	*p*-value
Memory (% of all participants)	N=52 (69%)	N=21 (60%)	N=11 (65%)	$\chi 2(2)$ 0.629
Attention	N=50 (67%)	30 (86 %)	N=10 (59%)	Fisher's exact 0.052
Executive functions	N=37 (48%)	N=17 (49%)	N=5 (29%)	$\chi 2(2)$ 0.334
Fatigue	N=16 (62%)	N=6 (17%)	N=4 (24%)	Fisher's exact 0.831
**Neuropsychological test, N**	Completers: mean (SD), N	Drop-outs:rivin mean (SD), N	Non-starters: mean (SD), N	ANOVA *p*-value
Logical memory (WMS), raw scores (max 50 points), N=84	21.8 (6.4), N=48	24.0 (8.5), N=26	20.8 (8.6), N=10	0.369
Logical memory (WMS), delayed, raw scores (max 50 points), N=89	20.0 (6.1), N=52	21.5 (7.7), N=27	17.5 (10.4), N=10	0.314
Word list, ten words x 4 (max 40 points), N=108	29.8 (5.4), N=65	30.6 (5.3), N=30	30.9 (6.1), N=13	0.737
Word list, delayed (max 10 points), N=108	6.9 (2.2), N=65	6.9 (2.9), N=30	6.7 (2.3), N=13	0.937
Word fluency, words starting with letter K per minute, N=115	16.7 (6.6), N=70	15.3 (5.9), N=32	18.9 (8.9), N=13	0.272
Word fluency, animals per minute, N=122	19.5 (6.8), N=73	19.1 (6.6), N=33	20.2 (8.0), N=16	0.874
TMT-A, time (s), N=124	45.9 (23.4), N=74	50.1 (27.9), N=34	40.8 (25.7), N=17	0.458
TMT-B, time (s), N=125	114.2 (58.4), N=74	115.9 (72.4), N=34	105.2 (79.8), N=17	0.850
Finger tapping in 10s, dominant, N=98	52.3 (9.4), N=62	50.3 (8.6), N=21	52.5 (8.9), N=15	0.676
Finger tapping in 10s, non-dominant, N=98	47.7 (10.1), N=62	42.7 (10.8), N=21	50.1 (7.0), N=15	0.062

WMS: Wechsler Memory Scale; TMT-A: Trail Making Test part A; TMT-B: Trail Making Test part B
